# Assessment of the health care waste generation rates and its management system in hospitals of Addis Ababa, Ethiopia, 2011

**DOI:** 10.1186/1471-2458-13-28

**Published:** 2013-01-12

**Authors:** Mesfin Kote Debere, Kassahun Alemu Gelaye, Andamlak Gizaw Alamdo, Zemedu Mehamed Trifa

**Affiliations:** 1 Department of Epidemiology and Biostatistics, College of Medicine and Health Sciences, Arba Minch University, Arba Minch, Ethiopia; 2 Institute of Public Health, College of Medicine and Health Sciences, University of Gondar, Gondar, Ethiopia; 3 Arba Minch College of Health Sciences, Arba Minch, Ethiopia

## Abstract

**Background:**

Healthcare waste management options are varying in Ethiopia. One of the first critical steps in the process of developing a reliable waste management plan requires a widespread understanding of the amount and the management system. This study aimed to assess the health care waste generation rate and its management system in some selected hospitals located in Addis Ababa, Ethiopia.

**Methods:**

Six hospitals in Addis Ababa, (three private and three public), were selected using simple random sampling method for this work. Data was recorded by using an appropriately designed questionnaire, which was completed for the period of two months. The calculations were based on the weights of the health care wastes that were regularly generated in the selected hospitals over a one week period during the year 2011. Average generation indexes were determined in relation to certain important factors, like the type of hospitals (public vs private).

**Results:**

The median waste generation rate was found to be varied from 0.361- 0.669 kg/patient/day, comprised of 58.69% non-hazardous and 41.31% hazardous wastes. The amount of waste generated was increased as the number of patients flow increased (r_s_=1). Public hospitals generated high proportion of total health care wastes (59.22%) in comparison with private hospitals (40.48%). The median waste generation rate was significantly vary between hospitals with Kruskal-Wallis test (X^2^=30.65, p=0.0001). The amount of waste was positively correlated with the number of patients (p < 0.05). The waste separation and treatment practices were very poor. Other alternatives for waste treatment rather than incineration such as a locally made autoclave should be evaluated and implemented.

**Conclusion:**

These findings revealed that the management of health care waste at hospitals in Addis Ababa city was poor.

## Background

Medical waste (MW) refers to hazardous waste (HW) materials generated by healthcare activities, including a broad range of materials, from used needles and syringes to soiled dressing, body parts, diagnostic samples, blood, chemicals, pharmaceuticals etc. [1-6]. Exposure to infectious health care waste (HCW) can cause serious health problems particularly for waste collectors, hospital patients and healthcare workers [7,8]. Improper disposal of HCW can have a detrimental effect on the environment and the improper treatment of HCW in poorly designed incinerators causes the generation of hazardous pollutants [9,10]. According to the world health organization (WHO), HCW constitutes HW and non-hazardous wastes (non-HW), 10 - 25% of HCW is hazardous. HW consists of infectious materials such as contaminated blood and other body fluids, used containers and biological material, sharps, pharmaceuticals, pathological and chemicals and substances with high heavy metal content [11].

**Figure 1:**
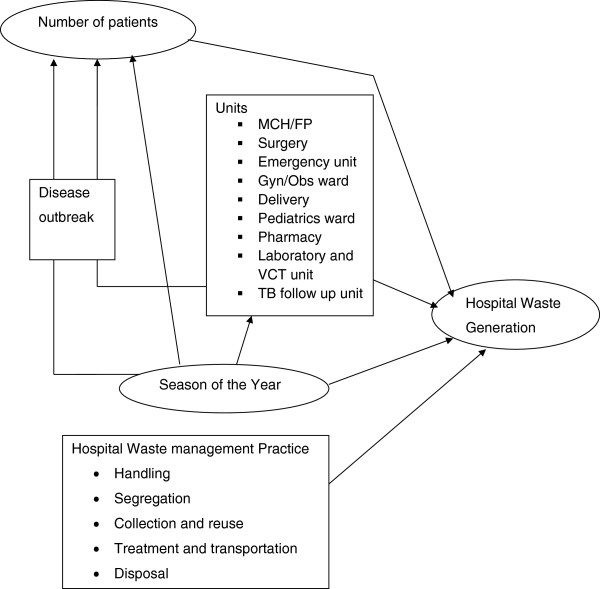
Factors that determine healthcare waste generation rate in Hospitals, Ethiopia.

HCW generation depends on numerous factors such as type of health care facilities (HCF), hospital specialization, available waste segregation options, seasonal variation, the number of hospital beds, and proportion of patients treated on a daily basis [11] (see Figure 1, which depicts the different factors affecting waste generation rates). According to Komilis et al., the mean unit generation rate of total MWs from HCFs in Greece was varied from 0.00124 kg/bed/day (private psychiatric hospitals) to 0.718 kg/bed/day (public university hospitals). There was a positive correlation between the total MW generation rates (kg/day) and the number of beds. No clear correlation was observed between the generation rates of the infectious/toxic and toxic medical wastes and the number of beds [12]. The generation rates of infectious wastes for nine general hospitals in Central Macedonia were varied from 0.51 to 1.22 kg/patient/day. The average quantity of infectious wastes produced by a general hospital is 198.3 kg/day. There is a linear correlation between the generated quantities of infectious wastes (kg/day) and the number of beds for all (twelve) government hospitals (r_s_ = 0.884) and for the general hospitals (r_s_ = 0.945) [13].

In Dhaka city 78 to 90% was general, 5-16% was infectious, 2- 6% was sharps and 1–3% was pathological [14]. In Bangladesh, a total of 2490 kg/day HCW (0.57 kg/patient/day, of which 0.21 kg/patient/day was HW and the rest 0.36 kg/patient/day was non-HW) was produced in Chittagong Medical College Hospital. The amount of HCW produced in the hospital was positively correlated with the number of beds (*r*_s_ = 0.79, *P *< 0.01) [15]. A study carried out in Iran and involved 14 hospitals indicated that the HCW generation rate was comprised of 51.6% of infectious waste, 47.2% general waste and 1.2% sharps [16]. A study done in 3 public hospitals in Agra city, India revealed that the average waste generation per day was found to be 25.3 kg at Lady Loyal Hospital, 482.9 kg at S.N. Medical College & Hospital and 500.5 kg at District Hospitals [17]. The generation rate in four hospitals of Nablus city, Palestine was between 0.33 and 0.84 kg/patient/day [18].

A study carried out in Dar es Salaam, Tanzania and involved 47 hospitals indicated that the total amount of HCW was 0.134 kg/patient/day (0.076 kg/patient/day HW and 0.058 kg/patient/day non-HW. Seventy percent of the waste was HW [19]. A study by Engdaw et al., in Gondar University Hospital (with 376 beds) in 2007 revealed that the mean HCW generation in the 7 days for general, infectious, sharp, pathological, and pharmaceutical wastes were 184±27.9, 128±27.3, 3±1.7, 18±5.4, and 6±5.2 respectively [20]. According to Haylamicheal et al., the total quantity of HCW generated in Hawassa city was a median of 226.9 kg/day. The quantity of waste per day generated increased as the number of outpatients flow increased but not statistically significant. The quantity of waste generated per day at public HCFs was more than at private HCFs and the percentage of HW generated was significantly more (p < 0.05) [2].

### Health care waste management

HCW management includes all activities involved in waste generation, segregation, transportation, storage, treatment and final disposal of all types of waste generated in the HCFs. MW handling is a hazardous activity which requires a high standard of training. It calls for specific training that depends on the nature of the work in the hospital, the hazards and possibility of worker exposure, and the responsibilities of individual workers [10]. The lack of segregation between HW and non-HW, an absence of rules and regulations applying to the collection of waste and the on-site transport to a temporary storage location, a lack of proper waste treatment, disposal of MW along with municipal garbage, insufficient training of personnel, insufficient personal protective equipment (PPE) and lack of knowledge about the proper use of such equipment are among the factors contributing to poor HCW management [17].

The MW should be segregated for collection by using colored bags and containers (plastic, metal or paper) [17]. A study in four hospitals of Nablus city revealed that segregation of all waste materials was not conducted according to definite rules and standards. None of them used color coding. This is not in accordance with the proposal by the WHO. The normal practice was to use any color available in the markets, which was normally black or blue, for both general and HW [18].

Although treatment technologies and disposal methods differ for each type of HCW, segregation at source into different categories reduces the management, operation and treatment costs along with the risk of infection with these contaminants [3]. Most African countries, waste disposal were reported to be problematic [21]. A study conducted in HCFs in Tanzania has shown that in Ilala only 54% and in Kinondoni only 10% of the surveyed HCFs have waste disposal areas. Some of the waste is disposed of in open areas and some into latrines and in rubbish pits [22]. A report by Making Medical Injections Safer (MMIS) project conducted in District health facilities in Ethiopia showed that the most common methods of MW disposal were open burning in a hole 54%, low-temperature incineration 52% and open-air burning on the ground 18% [23]. A study conducted in Sidama zone in 2004 revealed that 42.5% of the HCF used incinerators for disposing used needles and other sharps while the rest used open burning and other methods to dispose used needles and other sharps [24]. Management of HCW has received attention in many developing countries. However, in Ethiopia systematic and comprehensive studies on HCW management are lacking. There is not sufficient recorded information on HCW management methods and technologies and this fact hinders the planning for better management of HCW [1]. It is also necessary for Ethiopia to examine such HCW from broader perspectives from generation to collection, storage and disposal. Therefore, this study was conducted to evaluate the present HCW management practices in selected hospitals in Addis Ababa city and determine the daily quantity of waste generated and identifying factors affecting it and forward an appropriate HCW management approach for the hospitals in the city.

## Methods

### Study area

According to the 2009 health indicators, the total numbers of hospitals in Addis Ababa, the capital city of Ethiopia were 41 [25]. About ten of them are public and are managed under Addis Ababa City Administration Health Bureau and Federal Ministry of Health (FMOH) and the remaining thirty one are run by private investors and non-profit organizations [26].This study was carried out in five general and one specialized hospitals. The general hospitals were engaged in providing diagnostic and medical treatment to in-patients, while also providing other services, such as out-patient, laboratory services, pharmacy services, cafeteria, emergency, delivery, family planning (FP), reproductive health services, voluntary counseling and testing (VCT) services etc. Amanuel hospital (the only specialized mental hospital) had a total of 260 beds with an average patients flow of 394 patients/day providing cafeteria, emergency, general medicine, FP, laboratory, VCT, pharmacy, moods and in-patient services. The total number of beds in Zewditu hospital, Gandhi memorial hospital, Hayat hospital, Bethezata hospital and Saint Yared hospital were 215, 112, 70, 64 and 30 respectively. The daily average patients’ flow of all hospitals was 346 patients per day (range: 239–394). The patients flow per day at Bethezata hospital (341), a private hospital with long years of experience, was comparable with Zewditu hospital (378 patients per day) and Gandhi memorial hospital (350 patients per day). Saint Yared hospital had a low patients flow (239 patients per day).

A cross-sectional survey was carried out in six selected hospitals of Addis Ababa. The study on these hospitals was considered sufficient to evaluate the HCW management in the city. A similar assumption was followed by Khajuria and Kumar [17] and Issam et al. [18]. It was assumed that public hospitals would generate more HCW in the city than others mainly due to their relatively high outpatient flow, the hospitals were allocated disproportionately into private, from 31 [3], and public, from 10 [3], and were selected using simple random sampling technique. The study was conducted from March 1 to April 30, 2011 into two phases: The 1^st^ phase was focused on the MW management of the hospitals, and the 2^nd^ phase was focused on determining the amount of waste generated.

### Data collection

In accordance with the study protocol, which was approved by the Institutional Review Board of University of Gondar, FMOH and Addis Ababa City Administration Health Bureau, Verbal consent from each hospital’s head was obtained. Standardized questionnaire adapted from the WHO for HCW [27] was used and a survey based on observation and key informant interviews were performed by trained data collectors. Questions like materials used for waste collection, numbers of outpatients and beds, and PPE were included for this assessment. Weighting scale was used to quantify the amount of HCW generated. Waste was collected and measured daily for seven consecutive days to estimate the amount of waste generated. The waste was classified in each hospital as sharps, infectious, pathological, pharmaceutical and non-HW, and deposited into different colored and labeled puncture-proof plastic containers. The containers were emptied in the usual place that the hospital used for disposal after its weight were measured and recorded with recorded data format sheet every day at 8:00 A.M. One day: means in this work, 24 hours from 8:30 AM until it reaches the starting time. Patients: in this work are referred these outpatients.

### Statistical methods

The data was entered into EPI- INFO version 3.5.1 and exported to SPSS version 16 for analysis. The data was not found to be normally distributed and therefore medians and ranges were determined. The median quantity of HCW generated in the hospitals was computed. Significance testing was conducted using Spearman’s rank correlation coefficient (r_s_) for testing the bivariate associations between the total amount of waste and the number of patients and number of beds [28]. The total amount of waste and percentage of HW generated at public versus private hospitals were compared using the Mann–Whitney T test [28]. Patient flow, HCW generation rate and categories of HCW were compared using Kruskal-Wallis test [28]. The results on evaluation of waste management system were reported using different descriptive statistics. Extent of strength was presented using p-value; and r_s_ also reported. P-value ≤ 0.05 was used as a cut point to determine significance.

## Results

### Generation and classification of health care waste

**Figure 2:**
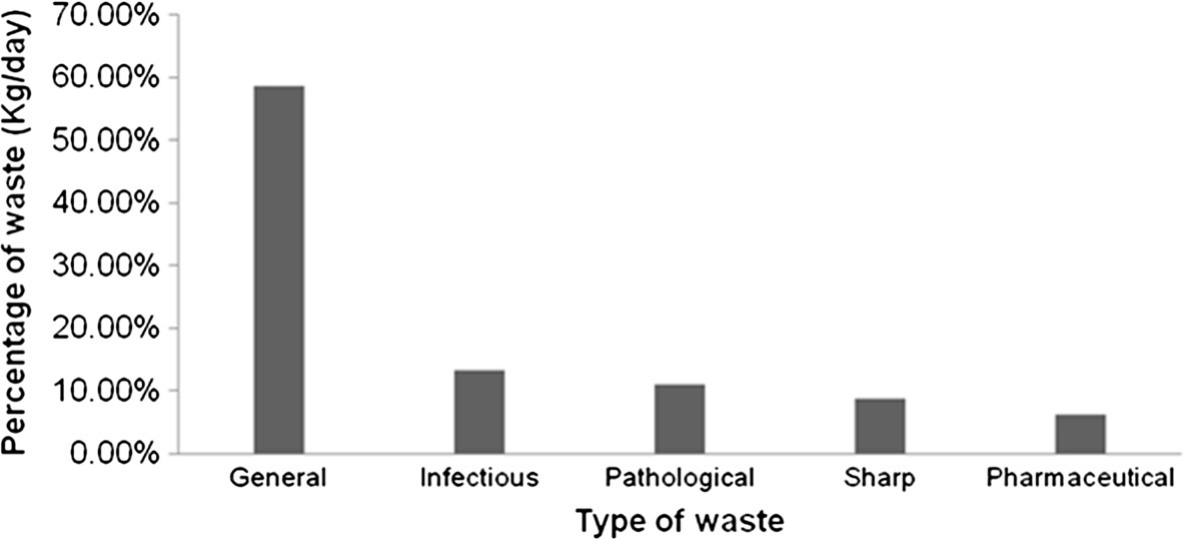
Percentage of pathological, infectious, pharmaceutical, general and sharp waste in the total HCW stream in all studied hospitals in Addis Ababa City, 2011.

The total quantity of HCW generated at all the hospitals was a median of 182.5 kg/day (range: 86.15–278.85 kg/day) (See Table 1). Two types of HCW were generated, namely non-HW (median: 58.69%, range: 46.89–70.49%) and HW (median: 41.31%, range: 29.5 – 53.12%), the majority of which was infectious (median:13.29%, range: 6.12-20.48%) and pathological waste (median:10.99%, range: 4.73-17.25%) and the rest sharps and pharmaceutical were (median: 8.74%, range:6.41-11.07%) and (median: 6.14%, range:3.54-8.73%) respectively (Figure 2). The median generation rate of infectious, pathological, pharmaceutical and sharps waste in each hospital was 25.50, 21.00, 12.00 and 15.13 kg/day, respectively. The highest generation rate of total HCW (0.668 kg/patient/day) was found in Amanuel specialized hospital while the lowest rate (0.525 kg/patient/day) was found in Bethezata hospital (Table 1). Amanuel hospital, a psychiatric hospital, had the highest non-HW generation rate (200.5 kg/day). Public hospitals generate a high proportion of total HCW (median: 59.22%) in comparison with the private hospitals, total (median: 40.48%). According to Table 2, the total quantity of HCW generated from public hospitals was significantly more (p < 0.05). But there was no a statistical significance difference between the amount of HW generated from public versus private hospitals (p-value = 0.51). With 5 degree of freedoms, there was a statistically significant difference for patient flow (X^2^=27.325, p-value = 0.0001), total amount of HCW (X^2^=30.65, p-value = 0.0001) and non-HW (X^2^=29.011, p –value = 0.0001) among the surveyed hospitals (See Table 3).

**Table 1:** Daily quantities of health care waste generation rates (median, IQR*) in the surveyed hospitals in Addis Ababa city, Ethiopia, 2011

**Name of hospital**	**kg/patient/day**						**kg/day**	
	**Non-HW**	**Infectious**	**Pathological**	**pharmaceutical**	**Sharps**	**Total HCW**	**Total HCW**	**percentages of HW**
Amanuel (public)	0.509 (±0.048)	0.098 (±0.033)	-	0.035 (±0.029)	0.039 (±0.018)	0.668 (±0.086)	263.50 (±33.75)	23.91%
Zewditu (public)	0.387 (±0.558)	0.116 (±0.013)	0.0741 (±0.019)	0.027 (±0.005)	0.070 (±0.021)	0.665 (±0.082)	251.2 (±31.10)	41.76%
Gandhi (public)	0.314 (±0.108)	0.037 (±0.011)	0.097 (±0.013)	0.037 (±0.007)	0.053 (±0.057)	0.531 (±0.159)	186.00 (±55.70)	40.86%
Bethezata (private)	0.297 (±0.027)	0.097 (±0.031)	0.051 (±0.006)	0.034 (±0.012)	0.043 (±0.010)	0.525 (±0.053)	179.00 (±18.00)	43.58%
Hayat (private)	0.373 (±0.096)	0.072 (±0.05)	0.084 (±0.030)	0.036 (±0.008)	0.056 (±0.054)	0.638 (±0.162)	167.10 (±73.60)	44.17%
Saint Yared (private)	0.357 (±0.085)	0.072 (±0.053)	0.054 (±0.022)	0.052 (±0.018)	0.0494 (±0.012)	0.579 (±0.071)	130.40 (±19.60)	34.59%

*IQR = Inter-quartile range.

**Table 2:** Comparison of public and private hospitals in Addis Ababa City for total and hazardous waste generated per day using Mann–Whitney T test, 2011

**Hospital type**	**Total HCW**		**HW**	
	**Mean rank**	**Sum of ranks**	**Mean rank**	**Sum of ranks**
Public	5	15	4	12
Private	2	6	3	9
P-value	0.05		0.51	

**Table 3:** Comparison of visitors, health care waste generation rates and categories of HCW using Kruskal-Wallis test among the surveyed hospitals, 2011

**Name of hospital**	**Mean Ranks**			
	**Patient flow**	**Total HCW**	**Non-HW**	**HW**
Amanuel	32.86	36.57	38.86	13.79
Zewditu	28.43	33.14	30.14	38.00
Gandhi memo.	28.86	19.00	17.43	20.07
Bethezata gen.	22.43	19.29	19.43	20.43
Hayat gen.	9.29	15.14	15.43	11.86
St. Yared gen.	7.14	5.86	7.71	24.86
Chi-square	27.325	30.650	29.011	20.431
P-valve	0.0001	0.0001	0.0001	0.001

According to Table 4, the Spearman’s rank correlation coefficient (r_s_) showed that there was a positive linear relationship between number of patients and quantities of HCW generation rates (p < 0.05). A positive linear relationship was also observed between total amount of HCW generated and number of hospital beds with (r_s_=0.943, p < 0.05).

**Table 4:** Correlation of visitors and quantity of waste generated in surveyed hospitals in Addis Ababa, Ethiopia, March 2011

**Name of hospital**	**Spearman’s rank correlation coefficient (r**_**s**_**)**
Amanuel Specialized Hospital	0.213
Zewditu General Hospital	0.393
Gandhi Memorial Hospital	0.607
Bethezata General Hospital	0.429
Hayat General Hospital	0.143
Saint Yared General Hospital	0.048
Total	1

### Health care waste separation, collection and transportation

Almost all of the hospitals reported that there was no segregation of waste into infectious, pathological and pharmaceutical, and had no separate bins for the collection of infectious waste. Sharps were stored in puncture proof safety box and only in one hospital the waste was segregated into pathological and non-pathological waste. None of the hospitals reported using a complete color coding system. It was observed that almost in all of the hospitals, non-HW was often mixed with infectious waste. Most of the HCW at the hospitals was found to be collected and transported in perforated plastic bins. HCW materials were collected and transported to a temporary storage area by waste handlers. In three hospitals, HCW materials were collected daily while the collection programme was irregular in the rest hospitals. In one hospital the waste was stored in temporary storage area up to one month before final disposal while in one other hospital stored for two weeks. The HW and non-HWs were mixed in the hospital’s temporary storage area and transferred to the disposal site. In three hospitals the hospital wards were near to the temporary storage site which is very hazardous to the health of the people.

### Treatment and disposal of health care waste

**Figure 3:**
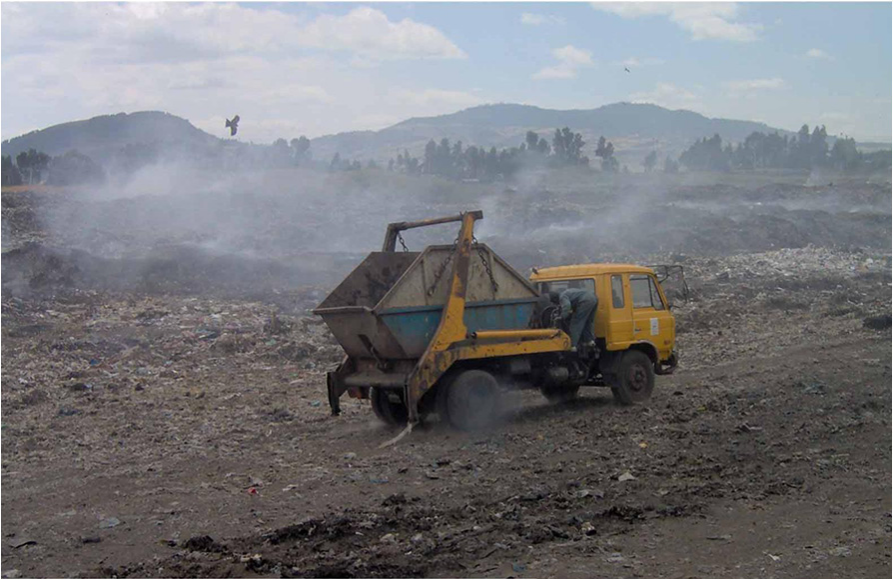
Kosha solid waste disposal unsanitary landfill site, Addis Ababa, Ethiopia.

Four of the surveyed hospitals disposed of their waste on-site in their own incinerators and one hospital disposed of at both off-site (non-pathological waste) and on-site (pathological waste) while the rest one hospital disposed of the waste at off-site (because the incinerator was not working at the time of data collection). Pre-treatment of highly infectious lab waste was not done in any of the hospitals. Only Zewditu and Gandhi hospitals disinfect sharps waste after use. Two studied hospitals that dispose their waste at off-site, the untreated hospital waste materials in the central storage area were finally loaded onto vehicles and transported to “kosha” unsanitary landfill site (Figure 3) for open dumping. The main HCW disposal mechanism in the studied hospitals was incineration, whereby, five of the hospitals had functional incinerators incinerating all the wastes together. Four of the hospitals used low combustion, single chamber, brick incinerators while one hospital used medium temperature incinerator as a treatment/final waste disposal method.

**Figure 4:**
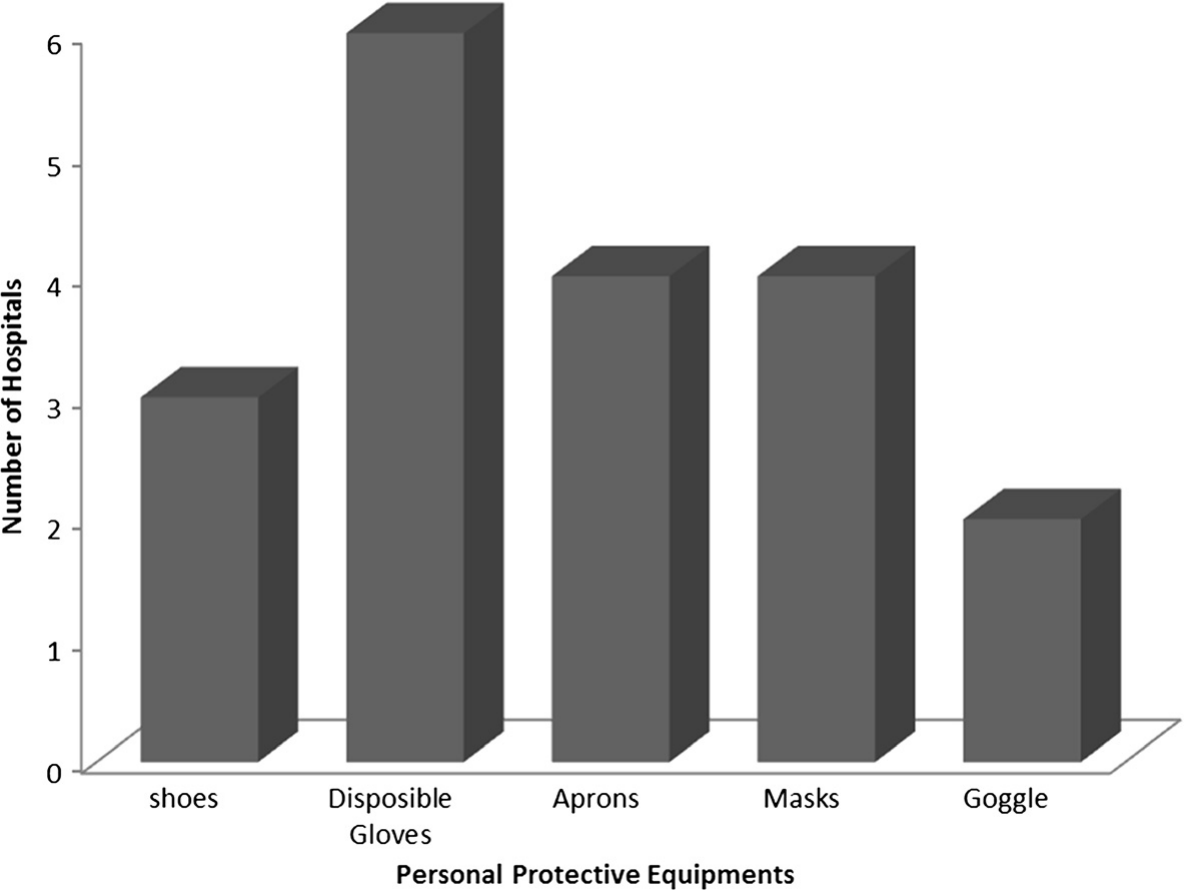
Distribution of the six hospitals and the protective gears used in the handling of health care waste by waste handlers in Addis Ababa, Ethiopia, 2011.

The majority of the surveyed hospitals incinerate their MW and most of the incinerators were present within the respective hospitals. Most of the incinerators have worn out chimneys or were made without chimneys, and most of the incinerators lack covers for the waste feeding door and in the ashes removing door. Two incinerators had not sufficient air inlets on the side and they did not locate downwind from the hospital. Furthermore, most [4] of the incinerators were small and not enough to handle the amount of MW generated (see Table 5). The main protective gears used in the surveyed hospitals were gloves, special dress and masks. Figure 4 shows the percentage distribution of the surveyed hospitals and the PPE used in the hospitals.

**Table 5:** Number and percentages of surveyed hospitals in Addis Ababa with respect to their incinerator specifications, 2011

**Incinerator specifications**	**Number of surveyed hospitals**	**Percentages of surveyed hospitals**
**Incinerator with no ash pits**	6	100%
**Incinerators with no sufficient air inlets on the side**	2	33.33%
**Incinerators not fenced**	4	66.67%
**Incinerators with worn out chimneys**	4	66.67%

## Discussion

The waste generated in healthcare activities is classified in to two: Non-HW and HW. Non-HWs are wastes that pose no risk of injury or infections. There are different estimates regarding to hazardous and non-HW constituents of HCW [1]. There was a statistically significant difference for total amount of HCW generated (X^2^=30.65, p-value=0.0001) and HW(X^2^=20.431, p-value=0.001) among the studied hospitals. The highest generation rate for total HCW was found at Amanuel hospital (0.668 kg/patient/day) and the highest amount for HW was found at Hayat hospital (0.265 kg/patient/day) and in contrast the lowest rate for total HCW and HW was found at Bethezata hospital (0.525 kg/patent/day) and Amanuel hospital (0.159 kg/patient/day respectively. In South Africa, the generation rate was computed to be 0.55 for Tshilidzini hospitals [11]. The generation rate indicates the types of services offered at Amanuel and Tshilidzini hospitals and suggest that they were different. The higher total HCW generation rates at Amanuel hospital were probably due to the fact that it is the only psychiatric and teaching hospital and thus serving a larger number of patients in comparison with other hospitals.

There was a statistically significant difference in generation rates of total HCW between public and private hospitals. The total HCW generated from public hospitals was significantly more (p < 0.05). This could be due to the differences in resource allocation and patient flow (there was statistically significance difference (X^2^=27.31, p-value=0.0001) between studied hospitals. The quantity of total HCW per day generated from hospital was increased as the number of patients (r_s_ = 1) and the number of beds (r_s_ = 0.943) increased with both being statistically significant (p < 0.05). This result was in agreement with a study conducted by Komilis et al., there was a positive correlation between the total MW generation rates and the number of beds [12]. This result was similar with the report of Haylamicheal et al. (2011), the amount of HCW was correlated with the number of beds (p < 0.05) [2]. Also, in Palestine, similar results were obtained by Issam et al. (2009) where the amount of HCW was positively correlated with the number of beds (*r*_s_ = 0.79 [18].

According to this work, the total HCW generation rate in the hospitals was varied from 0.361 to 0.669 kg/patient/day. This result was compared with the generation rates determined in other studies from different countries. For example, Komilis et al. reported that the mean unit generation rate of total MW in Greece was varied from 0.00124 to 0.718 kg/bed/day [12]. In Palestine the total amount of HCW generated was varied from 0.33 to 0.84 kg/patient/day [18]. The variation in total HCW generation among hospitals may be attributed to a variety of reasons such as the type of healthcare establishment, income level and welfare of patients and visitors and diversity of departments. Komilis et al., mentioned in their report that it is risky to make comparisons with MW records from other countries, due to the variability in the definitions and classifications of MW throughout the world [12].

The total HCW produced in the surveyed hospitals was found to be consists of 58.69% non-HW and 41.31% HW. According to Haylamicheal et al., 20–63.1% of the HCW was HW while the rest was non-HW [2]. This result was in agreement with the corresponding values, 74% non-HW and 26% HW, reported by Issam et al. [18]. The WHO has estimated the amount of HW in developing countries to be about 16%, 1% sharps and 15% infectious waste [11]. In the present work, however, the amount of HW was higher in the studied hospitals than the amounts recommended by the WHO. Observations during the present study showed that segregation of the HCW into the five defined categories was not implemented satisfactorily. Poor segregation practices at hospitals in Addis Ababa city in comparison with HCFs in other developing countries, as shown by Haylamicheal et al. [2], is the likely explanation for the relatively high proportion of HW found in the city. Lack of separation of infectious and non-infectious wastes at source increases the percentages of infectious wastes. Similar trends were reported by Haylamicheal et al. [2] and Azage [1] in their surveys. By designing and implementing an exact segregation programme in Addis Ababa city, the quantity of HW that needed to be treated would be reduced significantly. In addition, the monetary costs, public health risks and environmental pollution would be decreased similarly.

Most of the HCW at the hospitals was found to be collected in perforated plastic bins that are intended for use in administrative areas only. Waste was transported mostly in open plastic containers from the site of generation to the treatment area and all of the hospitals used safety boxes for collection of sharp wastes. It is better than the study done by Haylamicheal et al., Hawassa city, 67% of the HCFs did not use safety boxes for sharps [2]. This variation may be due to the risk of used needles and sharps related with improper collection might be given better attention by governmental health system and other Non-governmental organizations to reduce the transmission of HIV and other related disease, as shown by Azage [1], is the likely explanation.

The main disposal mechanism for HCW in the surveyed hospitals was incineration, five of them used incinerators (four of them used low combustion incinerators and one used medium temperature incinerators as a final disposal method). This was good compared with a report by MMIS project in Ethiopia, open burning in a hole 54%, low-temperature incineration 52% and open-air burning on the ground 18% [23]. This result was also good when compared with a study in Hawassa city (2008) which indicated 89% of the HCFs use low combustion incinerators and 11% of the HCFs uses open burning of the waste as a treatment method [2].

According to this work, it is worth mentioning here that the incinerators were not equipped with sufficient air-inlets, and so it endangered the health of the people living and working nearby. The final disposal method used in the surveyed hospitals was still poor when compared with other developed countries. This could be due to the use of low combustion single-chamber incinerators for the treatment of HCW was against the Stockholm Convection on persistent organic pollutants since such incinerators release dioxins/furans [2]. In Ethiopia the daily hospital records were not available; this finding didn’t account the bed occupancy (i.e., number of patients that occupy beds). So it is suggested that future research investigates the correlation of HCW generation rates with parameters other than the number of patients flow and the number of beds that the hospital have. Furthermore, the non-parametric analogue of multiple linear regression analysis was not used to control confounding during the data analysis stage. Composition of waste was not included in this specific study. There might be also observer bias during the assessment of HCW management system in this work.

## Conclusions

• The total HCW generation rates from hospitals in Ethiopia were varied from 0.361 to 0.669 kg/patient/day and the total HCW generated was found to consist of 58.69% non-HW and 41.31% HW.

• There was a statistically significant difference in generation rates of total HCW between public and private hospitals. The total HCW generated from public hospitals was significantly more (p < 0.05).

• The total HCW generated per day from a hospital was increased as the number of patients (r_s_ = 1) and the number of beds (r_s_ = 0.943) increased.

• Non-HWs, sharps, infectious waste, pharmaceutical and pathological wastes were the types of HCW generated in the surveyed hospitals.

• HCW were still handled manually and disposed of alongside non-HWs, thus creating a great health risk to waste handlers, the public and the environment.

• The waste separation and treatment practices of the hospitals were poor. Other alternatives for waste treatment rather than incineration such as a locally made autoclave integrated with a shredder should be evaluated and implemented.

## Competing interest

The authors declare that they have no competing interests.

## Authors’ contributions

MK participated in the coordination of the study, performed the statistical analyses and drafted the manuscript. AK participated in the design of the study and helped to draft the manuscript. AG and ZM conceived of the study, participated in its design and coordination, and helped to draft the manuscript. All authors read and approved the final manuscript.

## Pre-publication history

The pre-publication history for this paper can be accessed here:

http://www.biomedcentral.com/1471-2458/13/28/prepub
